# A Pyridazinone Compound for Effectively Treating Non-alcoholic Steatohepatitis by Targeting THRβ

**DOI:** 10.3389/fchem.2022.888587

**Published:** 2022-05-10

**Authors:** Hao Cheng, Xiao-Bo Wang, Ying Zhi, Bo Liu, Na Liu, Meng-Jun Li, Yan-Ling Mu

**Affiliations:** ^1^ School of Pharmacy and Pharmaceutical Sciences, Shandong First Medical University, Jinan, China; ^2^ Institute of Materia Medica, Shandong Academy of Medical Sciences, Jinan, China; ^3^ Key Laboratory for Biotech-Drugs Ministry of Health, Jinan, China; ^4^ Key Laboratory for RareUncommon Diseases of Shandong Province, Jinan, China

**Keywords:** pyridazinones, UPLC-MS/MS, pharmacokinetics, NASH, tissue distribution

## Abstract

Developing effective therapies and medicines to conquer nonalcoholic steatohepatitis (NASH) is of great significance for public health and is faced with a major challenge. The activation of the thyroid hormone receptor agonist THRβ could be regulated by target drugs that has brought huge potential to the treatment of NASH. In this work, pyridazinone compound YWS01125 was synthesized for the first time. In this study, an ultra-performance liquid chromatography–tandem mass spectrometry (UPLC-MS/MS) method for YWS01125 determination was established, and the pharmacokinetics of YWS01125 was evaluated. The half-life values (t1/2)of three different doses of YWS01125 was 189.12 ± 95.27, 152.64 ± 37.98, and 181.95 ± 64.25 min, respectively, and the tissue distribution studies demonstrated that YWS01125 was quickly distributed to various tissues. With successful application in the pharmacokinetics study of YWS01125, the UPLC-MS/MS method has shown characteristics of high sensitivity, rapidity, and good selectivity.

## Introduction

Nonalcoholic fatty liver disease (NAFLD) is characterized by heterotopic liver fat and steatosis (Dibba et al., 2018; Pierantonelli and Svegliati, 2019). NAFLD included a series of liver diseases, such as nonalcoholic fatty liver (NAFL), Nonalcoholic steatohepatitis (NASH), and liver fibrosis (Abdelbasset et al., 2019). With the increase of obesity and diabetes patients, the global incidence of NAFLD has shown an upward trend year by year. A few studies (Hannah et al., 2016; Friedman et al., 2018a; Kefala and Tziomalos, 2019; Janssen et al., 2020; Harrison et al., 2021) have reported that the global prevalence of NAFLD approximately accounted for 24–25% of the total population, which has been predicted to reach 28.40% in 2030.

The pathogenesis of NASH is very complicated. The “two-hit” theory is a widely accepted mechanism for NASH (Sumida et al., 2018) in which excess food intake leads to the accumulation of fat in the liver. However, with further research (Takahashi et al., 2014; Park et al., 2018; Peng et al., 2020), the “two-hit” theory can no longer reveal the complexity of this disease (Orci et al., 2016). Therefore, the “multiple-hit” theory proposed by Tilg and Moschen, 2010 (Younossi, et al., 2019) has gradually replaced the “two-hit” as the most widely accepted theory. The proposed mechanisms under this theory include disorders of lipid metabolism, lipid toxicity, insulin resistance, oxidative stress, mitochondrial dysfunction, changes in the intestinal microbiota, apoptosis, inflammation, and genetic susceptibility as the main components of this hypothesis (Janssen, et al., 2020). NASH can not only affect the functional structure of the liver but also increase the incidence of type II diabetes, cardiovascular disease, and chronic kidney disease (Castera et al., 2019).

Treatments for NASH can be divided into two main categories: drug therapy and nondrug therapy. Nondrug treatment is used to make appropriate adjustments in diet, physical exercise, and psychological stress relief, while drug therapy may include antioxidant therapy, lipid-lowering drugs, and weight-loss drugs; however, no highly effective drugs are available in the treatment of NASH in the present.

**FIGURE 1:**
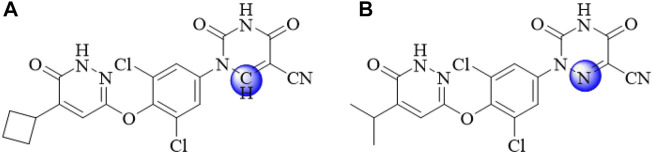
The chemical structures of **YWS01125 (A)** and **MGL-3196** **(B)**.

Therefore, it is extremely urgent to find drug targets to develop effective treatments. Thyroid hormone receptor agonist THR (namely, T3 and T4) includes two subtypes: THRα and THRβ. THRα is highly expressed in the skeletal muscle and heart, mainly regulating the heart rate, while THRβ is highly expressed in the liver, kidneys, and pituitary gland, mainly regulating the release of thyroid stimulating hormone (Younossi, 2019). THRβ selective agonist improves blood lipids, reduces Low density lipoprotein cholesterol (LDL) cholesterol levels, increases High density lipoprotein cholesterol (HDL) cholesterol reuptake, and reduces plasma triglyceride levels. Due to some side effects of THRα, THRβ was selected for a series of studies. Clinical trials (Tacke, 2018) have found that THRβ has good lipid-lowering properties in animals and small clinical trials. **MGL-3196** (Figure 1B), a selective THRβ agonist, is currently being evaluated in phase 3 clinical trials. It has been reported that resmetirom reduced both low density lipoprotein and triglyceride effectively and showed a liver-specific distribution, endowing it with good safety and making it a promising compound in the treatment of NASH (Harrison et al., 2019).

With regard to the fact that the azauracil moiety is a privilege scaffold for selective THRβ activation, we designed and synthesized a series of pyrazinone compounds bearing azauracil structure or its bioisostere. As a novel bioisostere of cynoazauracils, uracil derivatives **YWS01125** possesses the best selectivity and activity for THRβ with low cytotoxicity and was selected as the lead compound. In the pharmacodynamic study, it showed good *in vivo* efficacy in rat, such as hepatoprotective effect in an acute liver injury model, lipid-lowering function on acute hyperlipidemia mice, and alleviation of NASH. Therefore, we further conducted pharmacodynamics and pharmacokinetics research of **YWS01125** (Figure 1A). Firstly, we detected the metabolization of YWS01125 based on ultra-performance liquid chromatography–tandem mass spectrometry (UPLC-MS/MS). Then, the pharmacokinetics and tissue distribution of **YWS01125** in mice were further studied. As far as we know, this is the first reported study on the synthesis, pharmacokinetics, and tissue distribution of a novel THRβ selective agonist after oral administration.

## Materials and Methods

### Chemicals and Reagents

Pyridazinones: **YWS01125** (purity of 98.83%) was supplied by the Institute of Materia Medica, Shandong Academy of Medical Sciences (Jinan, China); **MGL-3196** (internal standard, IS) was purchased from Selleck Corporation (Beijing, China). The structural equations of the two compounds are shown in Figure 1. Nicotinamide adenine dinucleotide sodium phosphate (NADP), glucose-6-phosphate disodium salt (G-6-P), and glucose-6-phosphate dehydrogenase (G-6-PDH) were purchased from Sigma (Sigma, Germany). The reagent was purchased from Sigma Company (St. Louis, MO) and was chromatographically pure. The rat and human liver microsomes were purchased from Red Company (Shanghai, China). HPLC-grade methanol was supplied by Thermo Fisher Scientific Co. Ltd. (Shanghai, China); HPLC-grade acetonitrile was offered by Thermo Fisher Scientific Co. Ltd. (Shanghai, China); HPLC-grade formic acid was provided by Tianjin Kermel Chemical Reagent Co. Ltd. (Tianjin, China); purified water used for the all analysis was employed by Wahaha Co. Ltd. (Hangzhou, China).

### Chemistry

**SCHEME 1:**
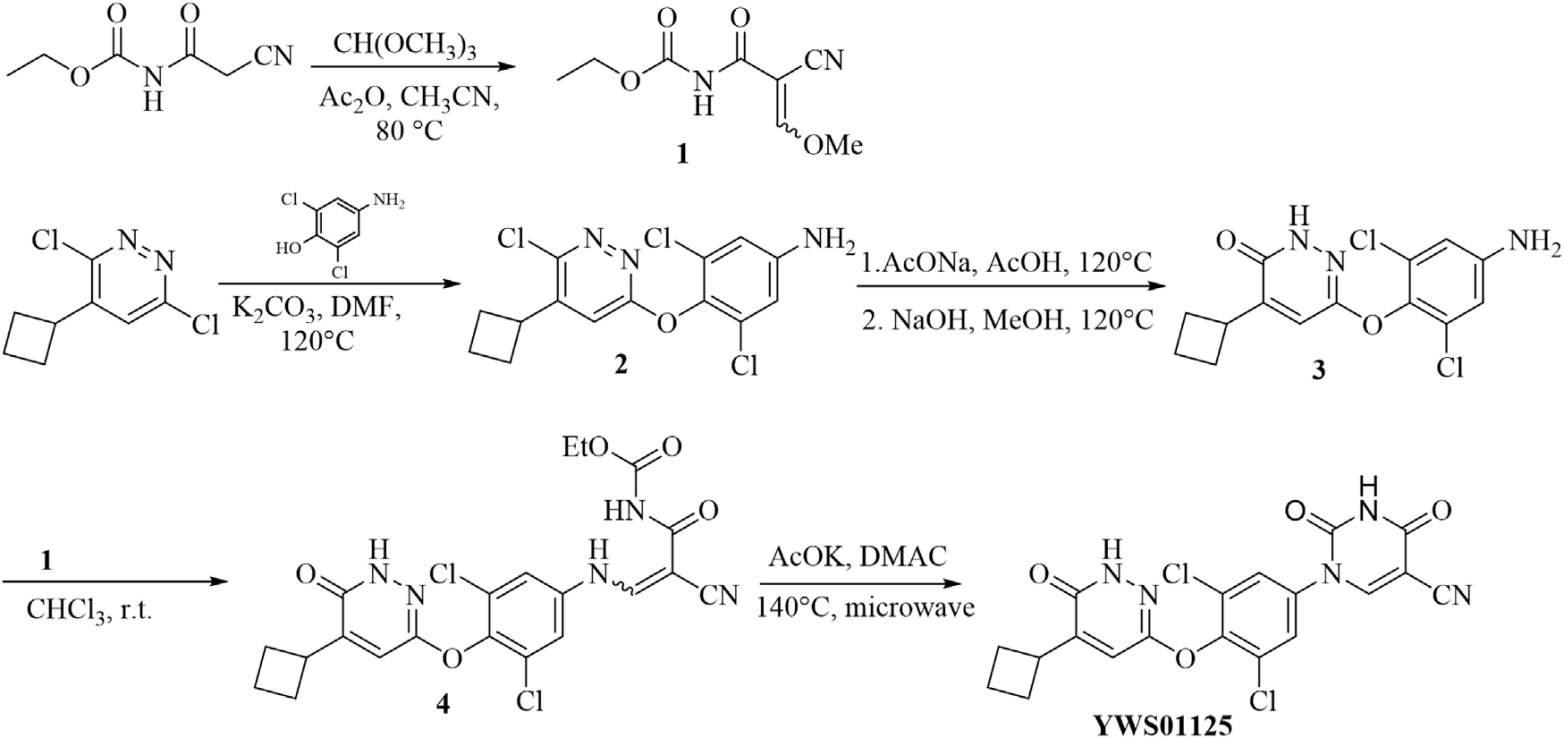
**(A)** CH(OCH_3_)_3_, Ac_2_O, CH_3_CN, 80°C; **(B)** 6-dichloro-4-cyclobutylpyridine, K_2_CO_3_, DMF, 120°C; **(C)** 1. AcONa, AcOH, 120°C; 2. NaOH, MeOH, 100°C; **(D)** ethyl (2-cyano-3-methoxyacryloyl) carbamate, CHCl_3_, r.t.; **(E)** AcOK, DMAC, 140°C, microwave.

The synthetic route of pyrazinone compound YWS01125 is as depicted in Scheme 1. Synthesis of 1-(3,5-dichloro-4-((5-cyclobutyl-6-oxo-1,6-dihydropyridazin-3-yl) oxy) phenyl)-2,4-dioxo-1,2,3,4-tetrahydropyrimidine-5-carbonitrile (YWS01125).

Synthesis of ethyl (2-cyano-3-methoxyacryloyl) carbamate (1).

Ethyl (2-cyanoacetyl) carbamate (3.12 g, 20 mmol), trimethyl orthoformate (3.5 ml, 40 mmol), acetic anhydride (20 ml), and acetonitrile (40 ml) were added to a 100-ml flask. The solution was stirred at 80°C for 4 h, and then the reaction stopped. The solvent was evaporated. Diethyl ether (50 ml) was added to the mixture, and the resulting suspension was kept at 2–8°C overnight. The suspension was filtered and rinsed several times with ether (3 × 50 ml) to give target compound **1** as a white solid (2.20 g, 56%). ESI m/z: C_8_H_11_N_2_O_4_ (M + H)+m/z = 199.1; molecular weight, 198.1780.

Synthesis of 3,5-dichloro-4-((6-chloro-5-cyclobutylpyridazin-3-yl) oxy) aniline (2).

The solution of 6-dichloro-4-cyclobutylpyridine (1.62 g, 8.00 mmol), 2, 6-dichloro-4-aminophenol (1.42 g, 8.00 mmol), and potassium carbonate (2.21 g, 16.00 mmol) in *N, N*-dimethylformamide (20 ml) was stirred at 120°C for 3 h. The reaction was stopped. An appropriate amount of diatomite was added into the sand core funnel, and the reaction solution was pumped and filtered to collect the filtrate. The filtrate was washed with 10% sodium chloride aqueous solution and saturated sodium chloride and was dried with anhydrous sodium sulfate. The solvent was evaporated to give a crude product which was purified by column chromatography (petroleum ether/ethyl acetate = 4/1) to obtain compound **2** as a light-brown solid (1.36 g, 50%). ESI m/z: C_14_H_13_Cl_3_N_3_O (M + H)+344.0; molecular weight, 344.6200.

Synthesis of 6-(4-amino-2,6-dichlorophenoxy)-4-cyclobutylpyridazin-3(2H)-one (3).

In a 100-ml round-bottom flask, the solution of compound **2** (1.36 g, 3.97 mmol), sodium acetate (1.30 g, 15.86 mmol), and glacial acetic acid (20 ml) was stirred at 120°C for 24 h. The reaction was stopped and the mixture cooled to room temperature. Then, 10 ml of water was added. The pH was adjusted to 9–10 with 1N HCl. The aqueous phase was extracted with ethyl acetate (25 ml × 3). The organic phase was combined, washed with brine, and dried over anhydrous sodium sulfate. The solvent was evaporated to give a crude product which was used directly in the next step without further purification.

The above obtained solid was dissolved in methanol (24 ml), and NaOH solution (1N, 24 ml) was added. The resulting mixture was stirred at 100°C for 24 h. The reaction was stopped. The solvent was evaporated. The suspension was extracted with ethyl acetate (25 ml × 3), and then the organic phase was combined, washed with brine, and dried over anhydrous sodium sulfate. The solution was concentrated under pressure and purified by column chromatography (petroleum ether/ethyl acetate = 2/1) to give compound **3** as a gray solid (0.57 g, 44%). ESI m/z: C_14_H_14_Cl_2_N_3_O_2_ (M + H)+326.0; molecular weight, 326.1770.

Synthesis of ethyl (2-cyano-3-((3,5-dichloro-4-((5-cyclobutyl-6-oxo-1,6-dihydropy-ridazin-3-yl) oxy) phenyl) amino) acryloyl) carbamate (4).

To a 10-ml reaction tube were added **1** (71 mg, 0.36 mmol), **3** (98 mg, 0.30 mmol), and chloroform (5 ml). The reaction mixture was stirred at reflux for 1 h and the reaction stopped. The mixture was then cooled to room temperature. The suspension was filtered and rinsed with ether. The filtrate cake was dried at 50°C. Compound **4** was obtained as a white solid (80 mg, 54%). ESI m/z: C_21_H_20_Cl_2_N_2_O_5_ (M + H)+492.1; molecular weight, 492.2130.

1-(3,5-dichloro-4-((5-cyclobutyl-6-oxo-1,6-dihydropyridazin-3-yl)oxy)phenyl)-2,4-dioxo-1,2,3,4-tetrahydropyrimidine-5-carbonitrile (YWS01125).

**FIGURE 2:**
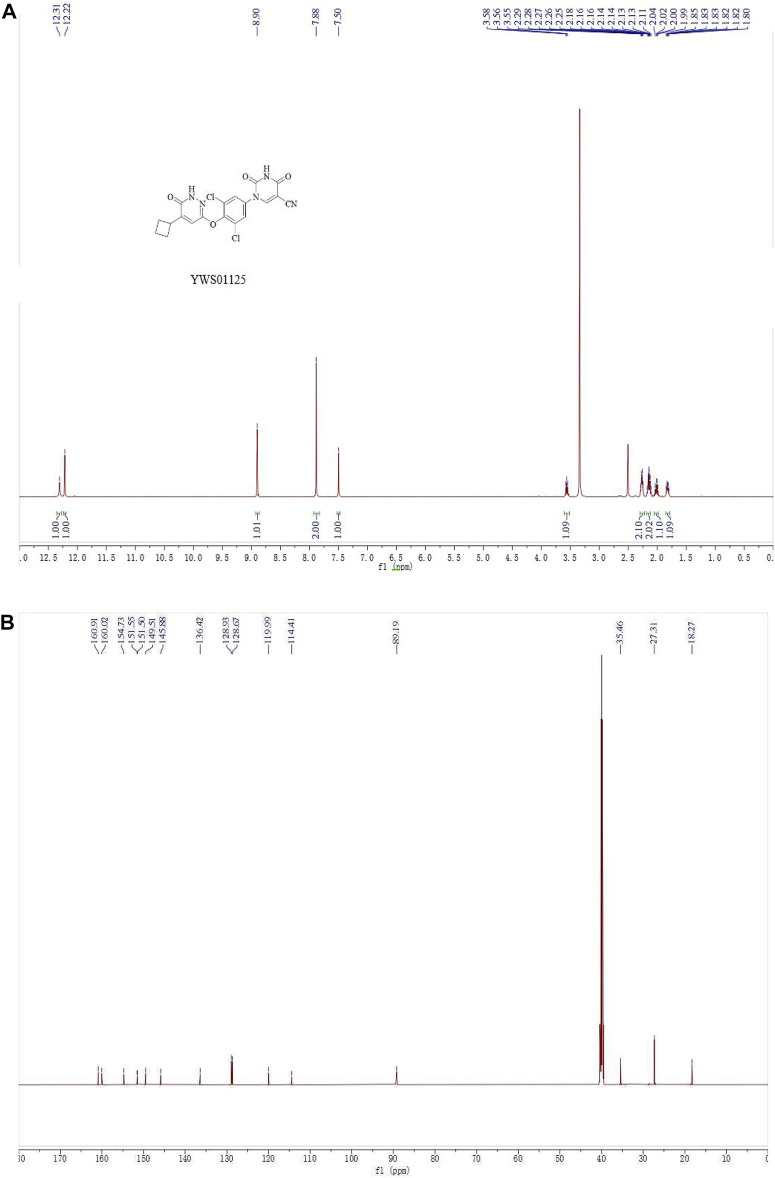
**(A)** ^1^H NMR spectra of **YWS01125**. **(B)** ^13^C NMR spectra of **YWS01125.**

Compound **4** (80 mg, 0.17 mmol) was added into a 10-ml microwave reaction tube, followed by the addition of potassium acetate (33 mg, 0.34 mmol) and N, N-dimethylacetamide (2 ml). The mixture reacted at 140°C for 30 min, then stopped. The reaction solution was diluted with ethyl acetate (20 ml), transferred into a 150-ml separating funnel, and diluted with water (10 ml). The organic phase was separated, washed with brine, and dried over anhydrous sodium sulfate. The solvent was evaporated to give a crude mixture which was purified by column chromatography (methylene chloride/methanol = 30/1 to 10/1) to give the target compound **YWS01125** as a light-yellow solid (37 mg, 52%). 1H NMR (600 MHz, DMSO-d6) δ 12.31 (s, 1H), 12.22 (s, 1H), 8.90 (s, 1H), 7.88 (s, 2H), 7.50 (s, 1H), 3.59–3.53 (m, 1H), 2.30–2.24 (m, 2H), 2.19–2.10 (m, 2H), 2.07–1.97 (m, 1H), 1.85–1.80 (m, 1H) ppm; 13C NMR (150 MHz, DMSO-d6) δ 160.9, 160.0, 154.7, 151.5, 151.4, 149.5, 145.9, 136.4, 128.9 (2C), 128.7 (2C), 120.0, 114.4, 89.2, 35.5, 27.3 (2C), 18.3 ppm; ESI-HRMS m/z calculated for C_19_H_14_Cl_2_N_5_O_4_ (M + H)+446.0423; molecular weight, 446.0420 (Figure 2).

### Animals

A total of 60 male C57BL/6J mice, weighting 23 ± 5 g, were obtained from Jinan Peng Yue Experimental Animal Breeding Co. Ltd. (Jinan, China). The permit number was SCXK (Lu) 20190003. The mice were allowed free laboratory sterile feed and sterile water and were housed in controlled environment conditions since at least 1 week before the experiment. The adjustable temperature was set at 20 ± 5°C and the moisture was 55 ± 15%, with a 12-h dark–light cycle. The tested mice were carried out according to the National Institute of Health Guideline for the Care and Use of Laboratory Animals, and performed by the Animal Ethics Committee of Institute of Materia Medica, Shandong Academy of Medical Sciences (Jinan, China). Before the pharmacokinetic study, all experimental mice had fasted for 12 h with free drinking water.

### Preparation of Stock Solution, Calibration Standard, and Quality Control Samples

The stock solution of **YWS01125** was prepared by accurately weighing a lot of the medicine, then dissolving it in methanol, yielding a final concentration of 1 mg/ml, and storing at 4°C. It was gradually diluted before use into a series of working solutions at concentrations of 4–400 ng/ml with acetonitrile. **MGL-3196** powder was prepared as a 1 mg/mL internal standard stock solution and stored in refrigerator at 4°C. Immediately before use, use chromatographic acetonitrile to dilute step by step to prepare an internal standard solution with a concentration of 2 ng/ml **MGL-3196**, and placed in 4°C standby. These standards of the quality control (QC) samples were prepared by diluting the stock solution of **YWS01125** to the blank plasma at low, medium, and high concentrations of 10, 50, and 300 ng/ml, respectively, and by depositing them at 4°C until analysis. All solutions were kept at 4°C before use.

### High-Performance Liquid Chromatography Conditions

Hypersil GOLD C18 column (4.6 × 150 mm, 5 μm, Thermo). A 0.025% ammonia aqueous solution in water (solvent A) and acetonitrile (solvent B) were used as mobile phases with gradient elution. The gradient was as follows: 0.01 min, 15% B; 5 min, 20% B; 12 min, 80% B; 16 min, 80% B; 16.01 min, 15% B; 19 min, 15% B; and 19.01 min, stopped. The timing interval was 19 min, and the flow rate was set at 1 ml/min.

### Chromatographic and Ultra-Performance Liquid Chromatography–Tandem Mass Spectrometry Conditions

Sorvall Biofuge Stratos high-speed refrigerated centrifuge was bought from the Thermo Fisher Scientific Co. Ltd. (Shanghai, China); EL204 electronic balance was selected from Shanghai Mettler-Toledo Instruments Co. Ltd. (Shanghai, China); IKA Vortex Genius 3 Vortex Mixer was purchased from Aika Instrument Equipment Co. Ltd. (Guangzhou, China); and the test was carried out using an AB SCIEX™ 5500 Q-Trap^®^ mass spectrometer (Applied Biosystems, United States) coupled to a Shimadzu Prominence UPLC (ultra-performance liquid chromatography, Shimadzu, United States) system. It was equipped with a binary pump, a degasser, an auto sampler, and an automatic thermostatic column compartment. The column was utilized from Agela Technologies UHP Innoval C18 (2.1 × 50 mm, 1.9 μm; Agela Technologies; Made in China). The whole process with a gradient elution program is as follows: 0.00–0.50 min, 5% B; 0.50–1.50 min, 90% B; 1.50–3.00 min, 90% B; 3.00–3.10 min, 15% B; and 3.10–5.00 min, 15% B. The mobile phase was made up of 0.10% formic acid in water (A) and acetonitrile (B), and the velocity of flow was 0.50 ml/min. The auto sampler and column temperature were set at 15 and 40°C, respectively. The injection volume was 1 μl. The Q-Trap mass spectrometer was operated with positive electrospray ionization source (ESI) and scanned using the multiple-reaction monitoring (MRM) mode. The Analyst^®^ software version 1.6.3 (Applied Bio-systems, AB SCIEX™, United States) was used to control the UPLC-MS/MS system. The best of the **YWS01125** parameters was curtain gas, 35 psi; collision gas, medium; ion spray voltage, 5500 V; temperature, 500°C; ion source gas 1, 50 psi; and ion source gas 2, 50 psi. The quantitative analysis ion pairs of **YWS01125** and **MGL-3196** (IS) were m/z 446.2 → 375 and m/z 435.2 → 182, individually. The suitable collision energy (CE) of **YWS01125** and IS was 42 and 50 eV, respectively, and the decluttering voltage (DP) was 160 and 180 V, respectively.

### Sample Preparation

The frozen mice plasma samples and tissue homogenates were thawed at room temperature, vortexed for 1 min, then taken out in 10-μl aliquots in a 1.50-ml centrifuge tube, and this was then mixed with 1 μl of standard solution and 50 μl of internal standard (IS) solution, which was then dissolved and diluted with acetonitrile. Subsequently, it was vortexed for 3 min and at 14,000 rpm centrifugation for 5 min. The adopted 1 μl of the supernatant was used for sample injection analysis.

### Method Validation

The validation was carried out by the US Food and Drug Administration bioanalytical method guidance (Bioanalytical Method Validation, 2018; US Food and Drug Administration, 2018; Chen et al., 2019), namely evaluating selectivity, linearity, accuracy and precision, recovery, matrix effect, and stability. Selectivity was assessed by contrasting the normal blank plasma samples' and analyst plasma samples' chromatograms from six different specimens. Linearity was appraised by different concentrations of the standard medicated plasma samples for 3 days continuously. The peak area was recorded (Booth et al., 2015), and weighted (1/x^2^) least-squares analysis was used to acquire the standard curve equations. The accuracy and precision were assessed by three different (low, medium, and high) QC samples by diluting with acetonitrile; each concentration had six samples and was continuously accessed for 3 days, and the accuracy, intra-day precision, and inter-day precision were obtained. Recovery was evaluated by comparing the peak area of protein precipitated with acetonitrile first and finally using it to calculate the recovery of the analyst. The matrix effect was measured by comparing the analyst peak area with the saline solution samples and QC samples at the three concentrations. The stability was assessed by defining the three QC levels in several different storage conditions: at room temperature for 6 h, injector stability at 15 °C for a whole night, and after three freeze–thaw cycles.

### Stability of Liver Microsomes Study

The incubation system was carried out in 0.05 M Tris/KCl buffer (PH = 7.4) and included the following: pyrazinone compounds dissolved with dimethyl sulfoxide (DMSO), mouse liver microsomes and human liver microsomes at 0.50 mg/ml, 1.30 mM nicotinamide adenine dinucleotide sodium phosphate (NADP), 3.30 mM glucose-6-phosphate disodium salt (G-6-P), 0.40 units/mL glucose-6-phosphate dehydrogenase (G-6-PDH), and 3.30 mM magnesium chloride. The volume of the organic solvent did not exceed 1% of the reaction system. After preincubation for 3 min in an 850-rpm blender (37°C), liver microsomes were added to initiate the incubation reaction (37°C). The incubation time was 0, 30, 60, and 90 min. After incubation, an equal volume of cold acetonitrile was added to stop the reaction, and an internal standard (0.05 mg/ml acetaminophen solution) was added. The samples were centrifuged at 10,000 rpm for 10 min to precipitate proteins (Day et al., 2017; Manne et al., 2018), and the 10 µL of the supernatant was taken for HPLC analysis.

### Pharmacokinetic Study

A total of 18 C57BL/6J mice were given **YWS01125** by oral administration with a dose of 10, 20, and 40 mg/kg, respectively (Lally et al., 2019; Kelly et al., 2014; Motamed et al., 2017) (with the concentration of drug administration selected according to the study by Kelly et al.). The blood samples of 20 μl were collected and placed in heparinized tubes from the ophthalmic venous plexus before and at 0.03, 0.08, 0.16, 0.25, 0.50, 1, 2, 4, 6, 8, 12, and 24 h. The samples were immediately centrifuged at 14,000 rpm for 10 min at 4°C. The plasma was separated and stored below −80°C until UHPLC-MS/MS analysis.

### Tissue Distribution Study

A total of 24 male C57/BL6J mice were divided into 4 groups at random, with 6 mice in a group for tissue distribution at 0.25, 1, 6, and 24 h. They were given **YWS01125** after oral administration of 20 mg/kg, and then sacrificed at 0.25, 1, 6, and 24 h. Tissues from the heart, liver, spleen, lungs, kidneys, stomach, small intestine, colon, fat, muscle, testes, and brain were taken and rinsed with saline solution to remove the blood and connective tissues (Geng et al., 2015; He et al., 2017; Jiang et al., 2017), then put on the filter paper and weighed, quickly. Each tissue sample was homogenized by using five times of the tissue weight saline (w/v). All homogenates were stored at−80 °C until analysis.

### Statistical Analysis

In this study, the comparison of the pharmacokinetic data between the normal and experimental groups was done using IBM SPSS Statistics 22.0 (Statistical Product and Service Solutions, United States) with an independent-samples *t*-test. The value of *p* < 0.05 was considered statistically significant.

## Results and Discussion

### Stability of Liver Microsomes Study

**FIGURE 3:**
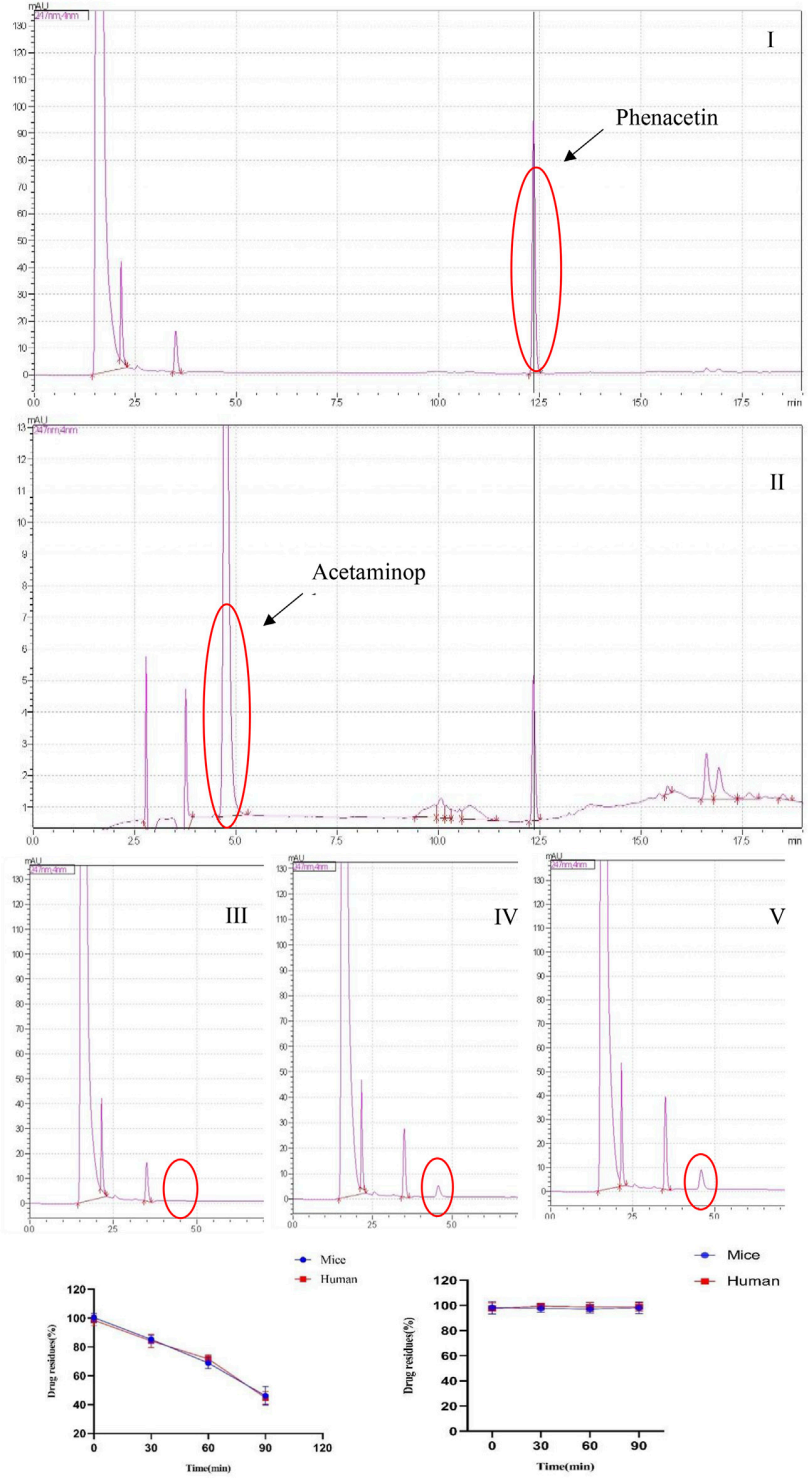
**(A)** Selected reaction monitoring chromatograms of phenacetin (I), acetaminophen (II), the peak of metabolite acetaminophen at 0 min (III), the peak of metabolite acetaminophen at 30 min (IV), and the peak of metabolite acetaminophen at 60 min (V) in the human liver microsomes. **(B)** Stability of phenacetin (I) and **YWS01125** (II) in mice and human liver microsomes.

The liver microsomal metabolic system was established. Phenacetin was used as a positive control, and its metabolite paracetamol were used for liquid phase analysis. The concentration of phenacetin peaked at 12.50 min, and its metabolite paracetamol peaked in about 5 min. In addition, phenacetin was added into the metabolic system and cultured for 0, 30, and 60 min. The results showed that its metabolite acetaminophen appeared in a time-dependent manner. However, **YWS01125** had no metabolites during the culture time in mice and human liver microsomes, suggesting that **YWS01125** was not metabolized in the liver microsomal system (Figure 3).

### Ultra-Performance Liquid Chromatography–Tandem Mass Spectrometry Chromatograms

**FIGURE 4:**
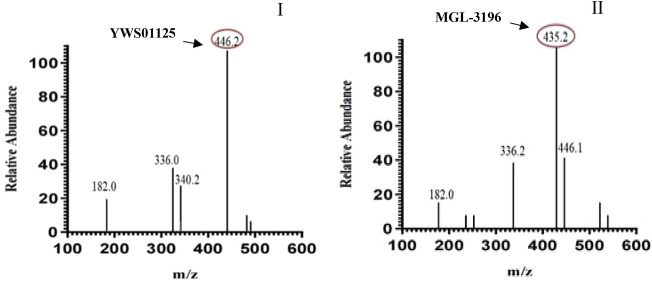
Positive-ion electrospray mass spectra of **YWS01125** (I) and IS (II).

**FIGURE 5:**
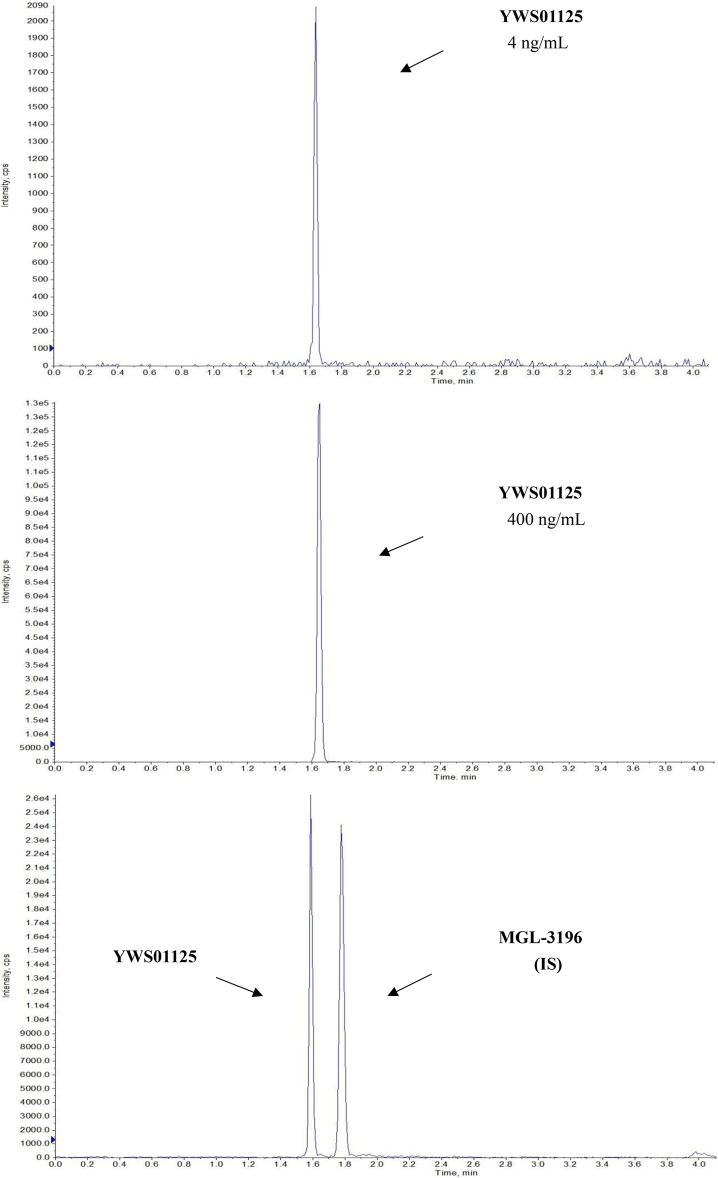
Representative LC-MS/MS chromatograms of **YWS01125** (4 and 400 ng/ml) and **MGL-3196** (IS) samples.

Under the above chromatographic conditions, the retention time for **YWS01125** and **MGL-3196** (IS) was 1.60 and 1.80 min, respectively. The production scan MS spectra of **MGL-3196** and **YWS01125** is shown in Figure 4. Figure 5 shows **YWS01125** of 4 and 400 ng/ml, and IS of 2 ng/ml UPLC chromatographic images. During optimization of the mass spectrometric parameters, a higher response was observed in the positive mode than in the negative mode for all of the analysts. The deprotonated precursor molecular ions (M − H)− were chosen to be monitored. The quantitative analysis was carried out in the MRM mode at m/z 446.2 → 336 for **YWS01125** and m/z 435.2 → 182 for MGL-3196 (IS). Meanwhile, the transitions were regarded as qualitative analysis. Finally, for **YWS01125**, the decluttering potential (DP), entrance potential (EP), cell exit potential (CXP), and collision energy (CE) were 160, 42, 15, and 28 V, respectively, while for MGL-3196, the DP, EP, CXP, and CE were 180, 50, 43, and 20 V, respectively.

### Method Validation

#### Specificity and Selectivity

**FIGURE 6:**
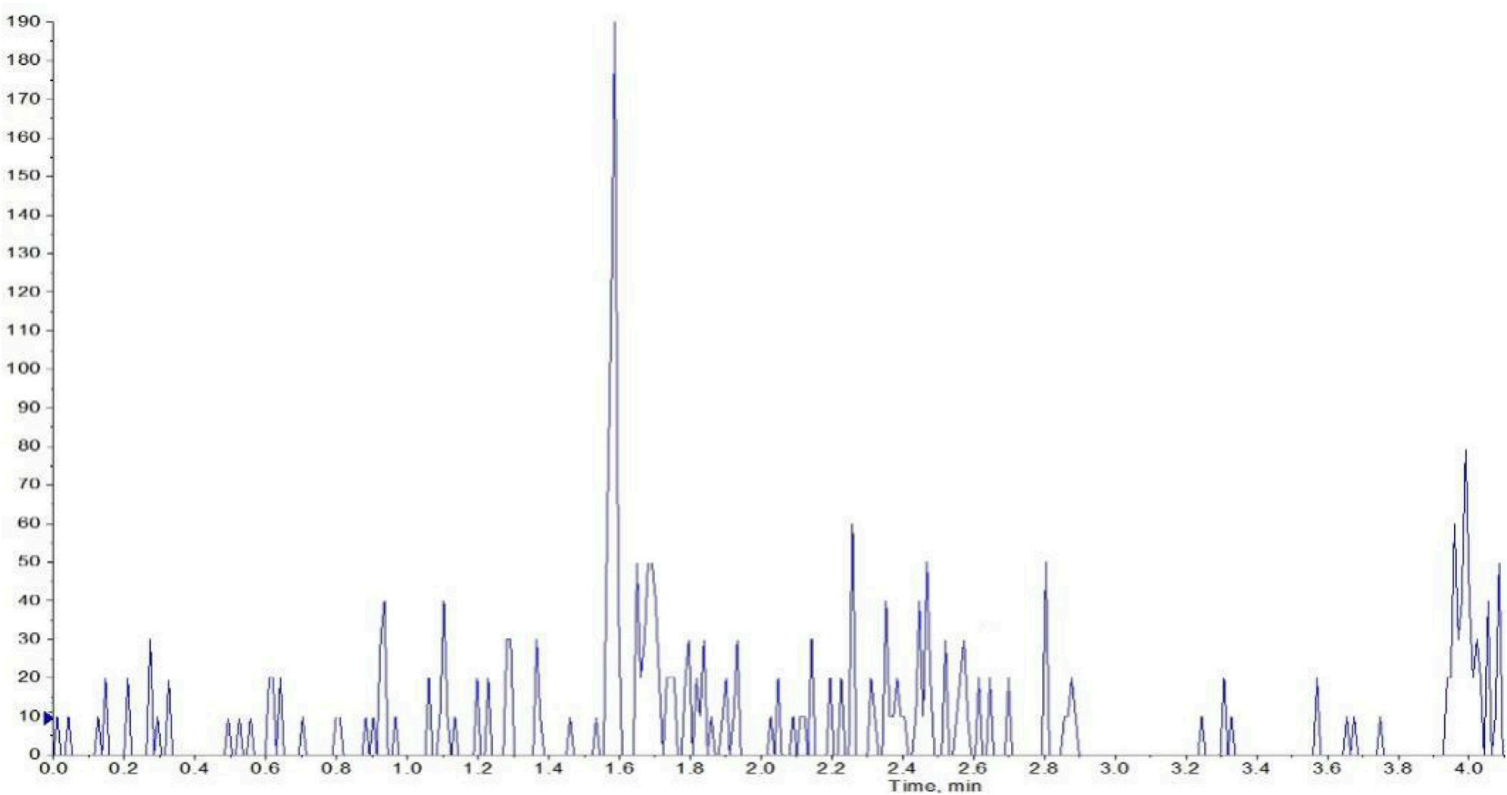
Chromatographic profile of the plasma of control mouse.

The selectivity of the method toward endogenous biological matrices was assessed with the plasma, heart, liver, spleen, lungs, kidneys, stomach, small intestine, colon, fat, muscle, testes, and brain from six mice. The retention times of **YWS01125** and IS were detected at 1.60 and 1.80 min, respectively (Figure 5). Comparing with the images of the control plasma samples (Figure 6) indicates that **YWS01125** and IS are well separated.

#### Linearity of Calibration Curve

**FIGURE 7:**
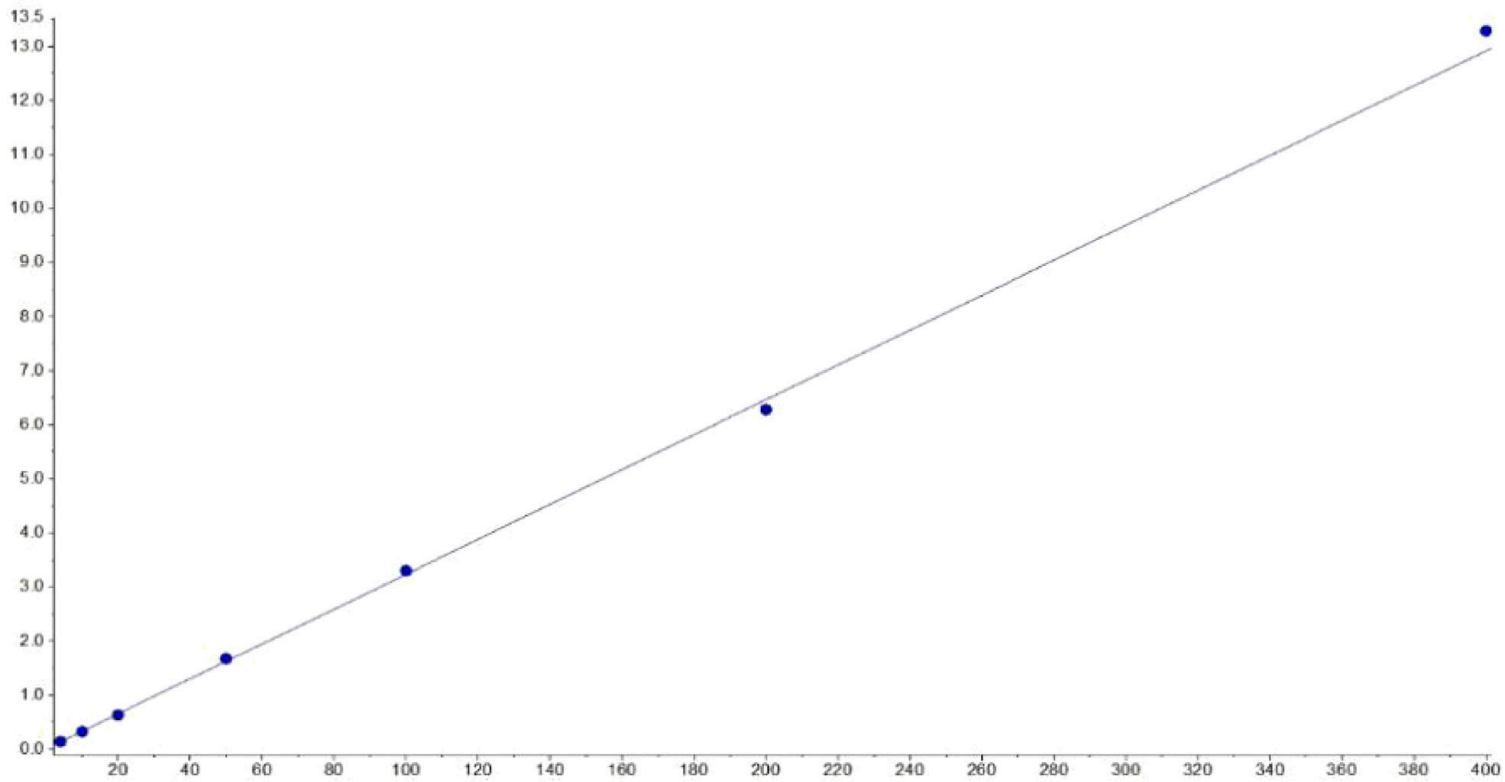
The linearity of the calibration curve.

The plasma calibration standard curve had a reliable reproducibility over the standard concentrations across the calibration range. The calibration curves' linear relationship was good in the range of 4–400 ng/ml, with a correlation coefficient (*r* ^2^) of 0.99 or better, and these were acquired on three consecutive days. The concentration of **YWS01125** as abscissa and the peak area ratio of **YWS01125** to IS as the ordinate implemented the regression equation with the weighted (1/x^2^) least squares method in acquiring the linearity of the calibration curve. The typical regression equations for the calibration curves were obtained as follows: Y = 0.0323 x + 0.00756 (r = 0.9994) for **YWS01125**. The The lower limit of quantification (LLOQ) with the relative standard deviation (RSD) ≤20% was found to be 4.00 ng/ml, with an RSD <9.24% (Figure 7 and Table 1) indicating of good sensitivity of the UPLC-MS/MS method.

**TABLE 1:** Precision and accuracy of UPLC-MS/MS for drugs in the mouse plasma (*n* = 6).

**Analyze**	**Added concentration (ng/ml)**	**Intra-day**			**Inter-day**		
		**Calculated concentration (ng/ml)**	**Precision (RSD, %)**	**Accuracy (RE, %)**	**Calculated concentration (ng/ml)**	**Precision (RSD, %)**	**Accuracy (RE, %)**
**YWS01125**	4	4.07 ± 0.22	5.45	1	4.09 ± 0.26	6.43	2.25
	10	9.34 ± 0.56	6.06	−6.6	10.34 ± 0.53	5.16	3.40
	50	47.66 ± 4.40	9.24	−4.68	48.95 ± 2.58	5.27	-2.10
	300	289.16 ± 12.76	4.41	−3.61	314.66 ± 10.85	3.45	4.88

#### Accuracy and Precision

The intra- and inter-day precision and accuracy data of **YWS01125** in the plasma are summarized in Table 1. The precision and accuracy of the developed method were validated by assaying the QC samples at four concentration levels (LLOQ, and low, medium, and high QC) in the plasma on three consecutive days (n = 6). In this assay, the intra- and inter-day precision (RSD, %) values were measured to be <9.24 and 6.43%, respectively, with the intra- and inter-day accuracy (%) in the range −6.60–4.88% in all biological matrices. The results met the requirements for the biological analysis with RSD ≤15% and RE within ±15%, suggesting a satisfactory precision and accuracy for the quantifications of all analysts in the mice plasma.

#### Extraction Recovery and Matrix Effect

The results of extraction recovery and matrix effects for **YWS01125** in biological matrices are summarized at three different QCs levels in Table 2. The recovery for each analysis at the three QC concentration levels range from 85.47 to 86.66% (n = 6) and the matrix effect from 110.00 to 115.00%, which indicates that the matrix effects under the current conditions are acceptable. The recovery of IS is 93.70% and the matrix effect of IS is 111.00%. These results show that the recovery of **YWS01125** in all biological matrices is consistent, reproducible, and independent; the values are all within the acceptable limit for extraction recovery. There is no significant matrix effect on analyses in this method. It is considered as negligible or insignificant matrix effect under the present conditions.

**TABLE 2:** The mean extraction recoveries and matrix effect of **YWS01125** and IS in the mouse plasma (n = 6).

**Analyze**	**Added concentration (ng/ml)**	**Recovery (%, mean ± SD)**	**Matrix effect (%, mean ± SD)**
**YWS01125**	10	86.66 ± 2.84	110 ± 0.08
	50	86.56 ± 2.21	112 ± 0.06
	300	85.47 ± 2.08	115 ± 0.12
IS	2	93.70 ± 2.84	111 ± 0.06

#### Stability

This experiment investigated the stability of **YWS01125** in the mouse plasma at different temperature conditions, namely the auto sampler (15°C) for 12 h of injector stability, room temperature for 6 h, and three freeze–thaw cycles. The results revealed RSD ≤ ± 15% and RE ≤ ± 15%, which has indicated that **YWS01125** maintains stability in the above three environments. The measured results of **YWS01125** are all within the acceptable limits during the entire process (Table 3).

**TABLE 3:** Stabilities of **YWS01125** in the mouse plasma (n = 6).

**Storage conditions**	**YWS01125**			
	**Added concentration (ng/ml)**	**Mean ± SD (ng/ml)**	**Precision (RSD, %)**	**Accuracy (RE, %)**
Injector stability	10	10.67 ± 0.52	4.91	5.00
	50	49.91 ± 2.15	9.24	−4.68
	300	295.17 ± 29.36	9.94	−1.60
Room temperature for 6 h	10	10.29 ± 0.82	9.06	1.20
	50	52.62 ± 1.73	4.29	−0.46
	300	299.33 ± 10.07	2.98	3.88
Three freeze–thaw cycles	10	9.45 ± 0.57	8.87	−1.80
	50	47.80 ± 3.96	4.80	−2.20
	300	289.17 ± 12.77	2.80	−2.66

### Pharmacokinetic Study

**FIGURE 8:**
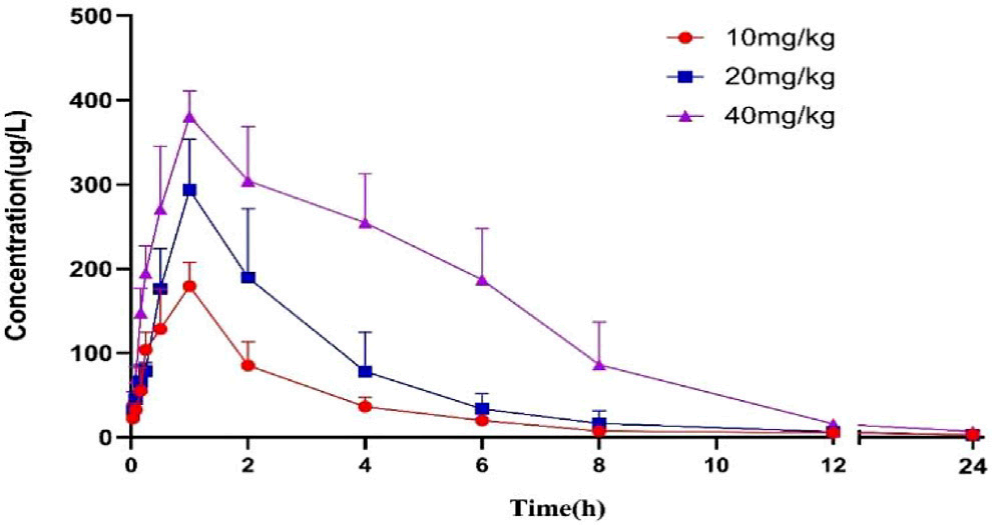
Mean plasma concentration–time curves after oral administration of **YWS01125** (10, 20, and 40 mg/kg) to C57BL/6J mice (mean ± SD, n = 6).

The validated method was applied to the pharmacokinetic study of **YWS01125** in normal C57BL/6J mice after oral administration, at doses of 10, 20, and 40 mg/kg suspensions with sodium carboxymethyl cellulose (CMCNa). **YWS01125** was measured in the plasma until the last blood collection time (24 h). The plasma concentration–time profiles of **YWS01125** following the single oral administration in C57/BL6J are presented in Figure 8. The main pharmacokinetic parameters of **YWS01125** were processed by a no compartmental model using the DAS 2.0 software package, and the pharmacokinetic parameters are summarized in Table 4. As can be seen, the peak concentration (C_max_) of each concentration of **YWS01125** in the mice plasma is 188.50 ± 30.03 ng/ml at 55.00 ± 12.25 min for 10 mg/kg, 293.42 ± 60.43 ng/ml at 60.00 ± 0.01 min for 20 mg/kg, and 381.58 ± 30.55 ng/ml at 55 ± 12.25 min for 40 mg/kg. From a comparison of the maximum plasma concentration (C_max_), the time to reach C_max_ (T_max_) and areas under the plasma drug concentration–time curve (AUC0–t and AUC0–∞) of **YWS01125** in the C57BL/6J mice were markedly increased, and there were statistically significant differences in these parameters (*p* < 0.05).

**TABLE 4:** Pharmacokinetic parameters of **YWS01125** after oral administration in the mice plasma (mean ± SD, n = 6).

**Parameter**		**Oral administration**		
		**10 mg/kg**	**20 mg/kg**	**40 mg/kg**
AUC0-t	mg/L*min	30921.38 ± 6261.12	54062.38 ± 18240.93	132269.96 ± 26808.23
AUC0-∞	mg/L*min	31378.67 ± 6086.08	54907.00 ± 19018.92	133818.83 ± 27275.44
MRT (0-t)	Min	205.41 ± 51.83	183.51 ± 23.10	274.15 ± 23.35
MRT (0-∞)	Min	242.69 ± 62.15	204.15 ± 25.50	296.57 ± 34.84^*^
t_1/2z_	Min	189.12 ± 95.27	152.64 ± 37.98	181.95 ± 64.25
T_max_	Min	55.00 ± 12.25	60.00 ± 0.01	55.00 ± 12.25
C_Lz/F_	L/min/kg	0.16 ± 0.02	0.25 ± 0.28	0.28 ± 0.05^*^
V_z/F_	L/kg	0.08 ± 0.04	0.09 ± 0.02	0.08 ± 0.03
C_max_	ng/mL	188.50 ± 30.03	293.42 ± 60.43	381.58 ± 30.55

*p < 0.05 compared with normal mice.

### Tissue Distribution

**FIGURE 9:**
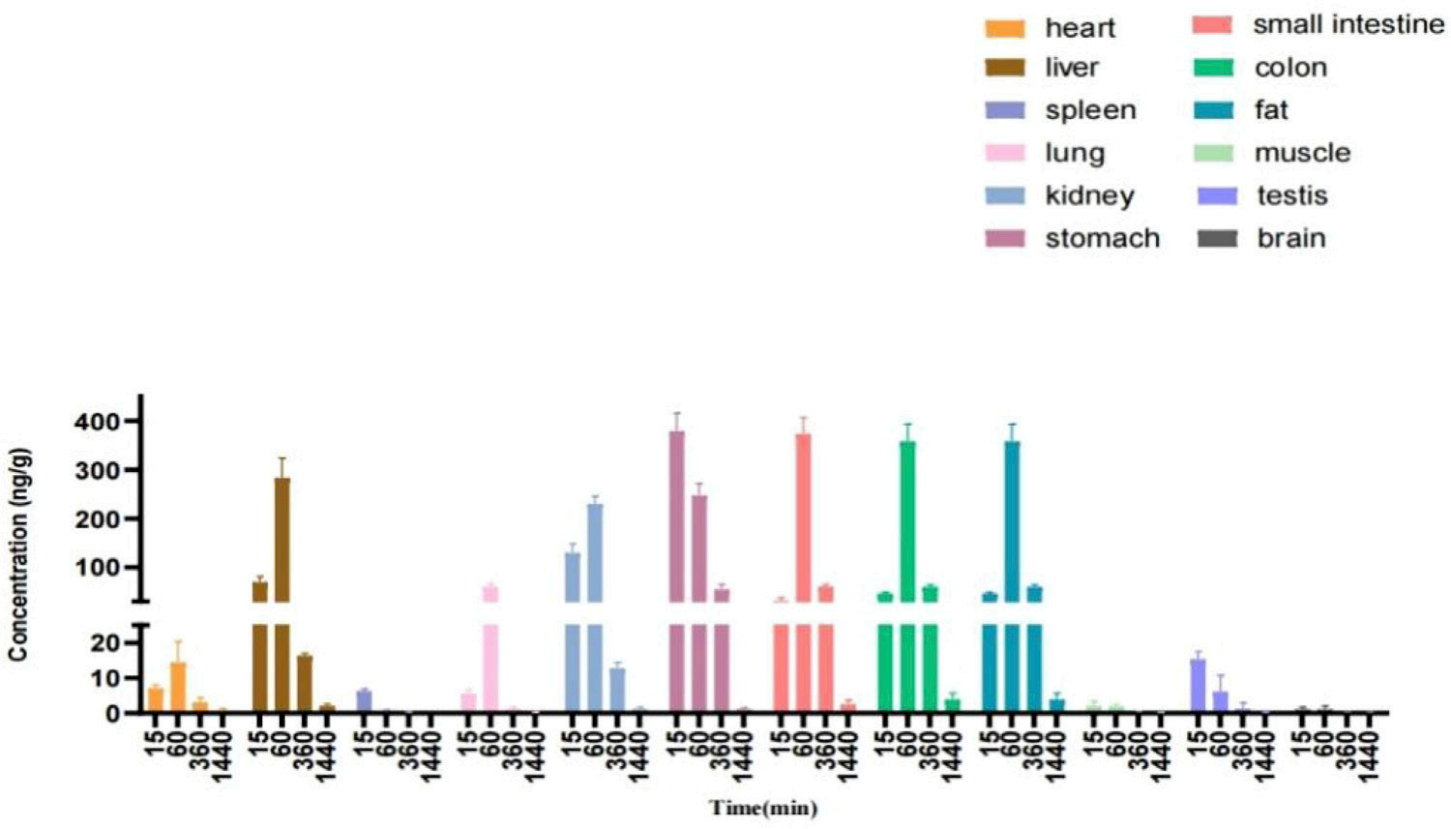
Tissue distribution of **YWS01125** (20 mg/kg) in C57/BL6J mice (mean ± SD, n = 6).

The tissue distributions of **YWS01125** were investigated in C57BL/6J mice at 0.25, 1, 6, and 24 h after oral administration. The examined organs or tissues included the heart, liver, spleen, lungs, kidneys, stomach, small intestine, colon, fat, muscle, testes, and brain. The results are presented in Figure 9. The linear ranges, correlation coefficients, weight factors, and regression equations for all analytes in the mouse plasma and tissue homogenates are shown in Table 5. The concentration of each tissue at 0.25 h is indicated as stomach > kidneys > liver > colon > small intestine > testes > heart > spleen > lungs > fat; concentration of each tissue at 1 h is small intestine > colon > liver > stomach > kidneys > lungs > heart > fat > testes; concentration of each tissue at 6 h is small intestine > colon > stomach > liver > kidneys > heart; and concentration of each tissue at 24 h is that the whole tissues were not detected (Table 6). After oral administration, the concentrations of **YWS01125** were not detected in the brain and muscle, suggesting that the blood–brain barrier may block the entry of the drug into the brain. The content of the compound in the stomach was much higher than that in any other organ. The highest levels were reached 0.25 h after oral administration, then decreased thereafter. For the heart, liver, kidneys, lungs, small intestine, colon, and fat, the highest level was reached 1 h after oral administration, and it reached the highest levels at 0.25 h in the spleen, stomach, and testes. These data show that **YWS01125** is quickly distributed throughout the mice body after oral administration; however, long-term accumulation might not be observed.

**TABLE 5:** Linear ranges, correlation coefficients, weight factors, and regression equations for all analytes in the mouse plasma and tissue homogenates.

**Analysts**	**Matrix**	**Linear range**	***r* ^2^**	**Weight factor**	**Regression equations**
**YWS01125**	Heart	4–400 ng/ml	0.9959	1/X^2^	Y = 0.0217 x + 0.0242
	Liver		0.9976		Y = 0.0226 x + 0.0563
	Spleen		0.9993		Y = 0.0237 x + 0.0223
	Lung		0.9960		Y = 0.022 x + 0.0311
	Kidneys		0.9988		Y = 0.0252 x + 0.00717
	Stomach		0.9989		Y = 0.0211 x + 0.0287
	Small intestine		0.9987		Y = 0.0223 x + 0.00278
	Colon		0.9989		Y = 0.0246 x + 0.0126
	Fat		0.9966		Y = 0.0244 x + 0.0337
	Muscle		0.9965		Y = 0.02 x + 0.00988
	Testes		0.9925		Y = 0.0244 x + 0.0423
	Brain		0.9986		Y = 0.0217 x + 0.00903

**TABLE 6:** Main organ tissue concentrations in mice after oral administration of **YWS01125**.

**Organs**	**15 min, mean ± SD**	**1 h, mean ± SD**	**6 h, mean ± SD**	**24 h, mean ± SD**
Heart	7.00 ± 1.05	14.41 ± 5.97	4.01 ± 1.27	ND
Liver	69.93 ± 11.25	283.67 ± 40.72	16.63 ± 1.72	ND
Spleen	6.26 ± 0.60	ND	ND	ND
Lungs	5.51 ± 1.22	60.76 ± 4.97	ND	ND
Kidneys	129.83 ± 18.31	230.50 ± 15.53	12.73 ± 1.65	ND
Stomach	378.67 ± 36.89	247.17 ± 24.96	54.49 ± 10.20	ND
Small intestine	31.30 ± 6.38	373.00 ± 33.88	61.03 ± 4.06	ND
Colon	46.70 ± 2.27	357.67 ± 35.31	60.23 ± 3.87	ND
Fat	9.37 ± 0.88	8.69 ± 1.45	ND	ND
Muscle	ND	ND	ND	ND
Testes	15.37 ± 2.15	6.08 ± 4.70	ND	ND
Brain	ND	ND	ND	ND

ND: not detected.

## Conclusion

In this study, a rapid, specific, and sensitive UPLC-MS/MS method for the determination of YWS01125 was developed and validated in the mouse plasma for the first time. In this method, carboxymethyl cellulose sodium suspension was used to dissolve drugs to maintain the homogeneity of the analytes. With the advantage of simple sample preparation procedure, short analysis time, and high sensitivity, the present method was successfully applied to the preliminary pharmacokinetic study of oral **YWS01125** in mice. This study demonstrated that as a precursor drug, **YWS01125** was absorbed very quickly and distributed extensively in C57BL/6J mice after oral administration. In addition, the UPLC-MS/MS method proved to be valuable in determining plasma **YWS01125**. *In vitro* experiments showed that **YWS01125** was stable and may not be metabolized in the mouse and human liver microsomes. Subsequently, the retention of **YWS01125** in the blood and its distribution in the various organs of mice were studied by *in vivo* experiments. It was found that **YWS01125** reached its highest concentration in the blood of mice at 1 h, and no drug distribution was detected in the muscle and brain of the mice group. In summary, **YWS01125**, a pyridazinone compound which we newly designed and synthesized, as an effective target, exhibited excellent activity targeting THRβ, and we studied its pharmacokinetics *in vivo* and found that it is a potential drug for the treatment of nonalcoholic fatty liver disease. The results are beneficial to further investigate the mechanism of YWS01125 and provide useful information for its clinical application.

## Data Availability

The original contributions presented in the study are included in the article/Supplementary Material, further inquiries can be directed to the corresponding author.
